# Comparative Analysis of the Oviducts in Wanyue Black Pigs with Different Parities Based on RNA-Seq

**DOI:** 10.3390/vetsci13010024

**Published:** 2025-12-25

**Authors:** Hanyu Zhou, Huibin Zhang, Ping Wu, Fang Tian, Jinyu Guan, Yifan Sun, Xiaodong Zhang, Zongjun Yin

**Affiliations:** 1 Anhui Province Key Laboratory of Local Livestock and Poultry, Genetic Resource Conservation and Breeding, College of Animal Science and Technology, Anhui Agricultural University, Hefei 230036, China; zhouhanyu27@163.com (H.Z.); wpwp8899@163.com (P.W.); 18391007746@163.com (F.T.); guanjy324@163.com (J.G.); syyyyyfan@163.com (Y.S.); 2 Anhui Key Laboratory of Livestock and Poultry Product Safety, Institute of Animal Husbandry and Veterinary Medicine, Anhui Academy of Agricultural Sciences, Hefei 230031, China; zhanghuibin1997@126.com

**Keywords:** parity, oviducts, Wanyue black pig, RNA-seq, PPI network analysis

## Abstract

This study investigates the molecular mechanisms underlying parity’s impact on sow reproductive function by comparing the transcriptome profiles of high-parity (9 parities) and low-parity (1 parity) Wanyue Black pigs. Oviduct tissues were collected and subjected to RNA-seq analysis. Differentially expressed genes (DEGs) were identified using the DESeq2 algorithm. A total of 4218 DEGs were detected, with 2421 up-regulated and 1797 down-regulated genes. Functional enrichment analysis using GO and KEGG revealed that these DEGs were significantly associated with reproductive pathways, including cilium movement, oocyte maturation, and steroid hormone biosynthesis. Protein–protein interaction (PPI) network analysis highlighted key genes such as HSD3B1 and DNAI1, which play central roles in the parity differences. The expression patterns of selected candidate genes were further validated by real-time quantitative reverse transcription polymerase chain reaction (RT-qPCR), and the results were consistent with the RNA-seq data, confirming the reliability of our findings. This study provides valuable insights into the molecular regulation of parity in sows and offers a theoretical basis for optimizing reproductive management and breeding strategies in pig production.

## Simple Summary

The present study aimed to elucidate molecular mechanisms underlying parity-mediated effects on sow reproductive function. While reproductive performance is parity-dependent, the oviduct’s specific contribution to this variation—particularly in indigenous breeds such as the Wanyue Black pig—has not been systematically characterized. Comparative RNA sequencing of oviduct transcriptomes between high-parity (9 parities) and low-parity (1 parity) sows identified 4218 differentially expressed genes. High-parity sows demonstrated upregulation of genes implicated in oocyte maturation and steroid hormone synthesis, thereby fostering a microenvironment conducive to early embryonic development. Conversely, low-parity sows exhibited elevated activity in genes controlling ciliary motility to optimize gamete transport. Key regulatory factors included *HSD3B1*, which mediates hormone synthesis, and *DNAI1*, which governs ciliary function.

## 1. Introduction

The reproductive performance of sows has a crucial impact on the production efficiency and economic sustainability of the pig industry [1]. Therefore, optimizing the reproductive efficiency of sows, especially improving their reproductive potential, has become one of the core goals of modern pig industry research. Parity, as a key factor influencing the reproductive function of sows, is gradually receiving more and more attention for its potential impact on reproductive performance [2]. Studies show that the number of live-born piglets varies depending on parity, exhibiting a trend of first increasing and then decreasing [3]. For example, Piñán J et al. [4] found that parity had a significant effect on the reproductive performance of Iberian sows. Sows with parity 2 to 4 showed the best reproductive performance, while sows with parity 5 to 10 showed poor reproductive performance. Similarly, Thomas et al. [5] showed that the total number of piglets born and the number of live-born piglets in multiparous sows were higher than those in primiparous sows. However, the stillbirth rate and mummified fetal rate of primiparous sows were slightly higher than those of multiparous sows. Parity not only has a significant effect on parity, but also alters a variety of reproductive behaviors. For example, high-parity sows exhibit stronger maternal protective behaviors, and there are large individual differences in the frequency and duration of lactation [6].

In addition, sows with different parities also have differences in immune transmission. Studies by Nuntapaitoon [7] and others have shown that the concentration of *IgG* in the colostrum of multiparous sows is higher than that of primiparous sows, and the concentration of *IgG* shows a significant upward trend with the increase in parity. There is a positive correlation between the concentration of *IgG* in colostrum and the concentration of *IgG* in piglet plasma, which means that piglets of primiparous sows may face a higher risk of death due to weak immune protection. However, the effect of parity on gene expression and its regulatory mechanism in the reproductive system of sows has not been fully and systematically elaborated, especially at the molecular biological level, and the specific regulatory mechanism of parity on reproductive performance is still lacking in-depth research.

The oviduct plays a crucial role in the reproductive process by providing an ideal biomechanical and biochemical environment for fertilization and early embryo development [8]. Studies by Teijeiro et al. [9] have shown that the oviduct plays an important role in the reproductive process by regulating gamete physiology, responding to gamete presence, supporting embryo development, storing and transporting gametes, and being regulated by hormones. Studies by López-Úbeda R et al. [10] have shown that the oviduct plays an important role in sperm selection, capacitation regulation, and protection, providing the necessary conditions for successful sperm fertilization and embryo development. Studies by Zhu M et al. [11] have shown that the oviduct plays an important role in transporting eggs, providing a fertilization environment, cilia movement, and sperm selection, and is an important component of the female reproductive system. In view of this, comparing the differences in the transcriptome of the oviduct of sows with different parities will provide important theoretical support for revealing the molecular mechanism of parity affecting the reproductive function of sows.

Wanyue Black pig is a new breed of high-fertility black pig developed by Anhui Agricultural University and germplasm protection enterprises. The lineage of Wanyue Black pig includes 37.5% of the Chinese native breed Huai Pig and 37.5% of the domestic breed Beijing Black Pig, and the remaining 25% comes from the imported breed Duroc. Although there have been preliminary studies on the reproductive performance of Wanyue Black pig, the systematic analysis of the reproductive performance differences in sows with different parity, especially the gene expression level of their oviducts, is still insufficient. Existing studies have mainly focused on the effect of parity on the reproductive performance of other pig breeds, but there is still a lack of related studies on this breed of Wanyue Black pig. Therefore, this study aims to systematically compare the transcriptome differences in the oviducts of high parity (9 parities) and low parity (1 parity) Wanyue Black pigs through RNA-seq technology, and to further explore the gene regulation mechanism of parity on the reproductive system of this breed of sows.

This study addresses a critical knowledge gap by investigating the oviductal transcriptome of the Wanyue Black pig, a newly developed breed that incorporates 37.5% Chinese Huai pig germplasm. Unlike conventional studies limited to commercial breeds, this work examines a tissue rarely explored in indigenous pigs, providing empirical evidence that parity significantly remodels gene expression patterns associated with oocyte maturation, steroid hormone biosynthesis (*HSD3B1*), and ciliary motility (*DNAI1*). The comparison of extreme parity groups (1 vs. 9 parities) reveals the oviduct’s active role in mediating parity-related reproductive performance rather than serving as a passive conduit. These findings elucidate tissue-specific mechanisms underlying parity effects and establish a molecular framework for optimizing breeding and reproductive management strategies in locally adapted pig populations.

## 2. Materials and Methods

### 2.1. Animal and Sample Collection

The animal experiments in this study were approved by the Institutional Animal Care and Use Committee of Anhui Agricultural University (Approval No. AHAU20231202). Six reserve sows of the Wanyue Black pig located in pig farms in Anhui Province were selected, including 3 high-parity black pigs (9 parities) and 3 low-parity black pigs (1 parity). All experimental animals were maintained under identical feeding and management conditions within the same farm to minimize environmental variation. The selected sows were in the weaning to estrus interval (WEI), a critical period for reproductive recovery. The mid-fallopian tube of the reserve sow was extracted, and excess adipose tissue was removed. The fallopian tubes were immediately frozen in liquid nitrogen and stored in an ultra-low temperature refrigerator at −80 °C until subsequent analysis was required.

### 2.2. RNA Extraction, Library Construction, and Sequencing

Total RNA was isolated from sow oviduct tissue using Trizol reagent (Invitrogen, Carlsbad, CA, USA) according to the manufacturer’s protocol. Briefly, the oviduct tissues were homogenized in Trizol reagent, followed by chloroform extraction and centrifugation to separate the aqueous phase. RNA was then precipitated with isopropanol and washed with 75% ethanol, resulting in purified RNA pellets that were dissolved in RNase-free water for further analysis. Following RNA extraction, sample integrity was evaluated via 1% agarose gel electrophoresis to assess potential degradation and contamination. RNA purity and concentration were measured using a NanoPhotometer^®^ spectrophotometer (IMPLEN, Westlake Village, CA, USA), while integrity and quantity were analyzed with an RNA Nano 6000 assay kit on a Bioanalyzer 2100 system (Agilent Technologies, Santa Clara, CA, USA). Only samples with an A260/A280 ratio between 1.8 and 2.0 and an RNA integrity number (RIN) ≥ 7.0 were proceeded to subsequent analysis.

The total RNA samples qualified for quality inspection were subjected to RNA library construction and sequencing. Polyadenylated RNA was isolated from total RNA using oligo(dT)-coupled magnetic beads and then fragmented in buffer at elevated temperature. First-strand complementary DNA (cDNA) synthesis was performed via random hexamer priming, followed by second-strand synthesis. The resulting double-stranded cDNA was purified with AMPure XP beads and subjected to end repair, A-tailing, and adapter ligation. The adapter-ligated fragments were PCR-amplified and purified using AMPure XP beads to generate the final sequencing library. After the library was constructed, Qubit 2.0 was used for preliminary quantification, and the library was diluted to 1.5 ng/μL. Then, the Agilent 2100 BioAnalyzer (Agilent Technologies, Santa Clara, CA, USA) was used to detect the insert size of the library. RT-qPCR was used to accurately quantify the effective concentration of the library to ensure the quality of the library. After the library passed the detection, DNB (DNA Nano Ball) was prepared, and then loaded onto the sequencing chip for sequencing using the MGI high-throughput sequencer.

### 2.3. Analysis of RNA-Seq Data

Raw reads were processed with SOAPnuke (v2.1.0) [12] (v2.1.0, https://github.com/BGI-flexlab/SOAPnuke, accessed on 15 October 2025) to remove low-quality reads, adapter-contaminated reads, and reads containing poly-N sequences. This meticulous step entails the elimination of reads that contain junctions, are of substandard quality, or contain poly N sequences. The clean reads were aligned to the porcine reference genome *Sus scrofa* (pig)-11.1(https://ftp.ensembl.org/pub/release-109/gtf/sus_scrofa/, accessed on 17 October 2025) using HISAT2 [13] (v2.1.0, https://github.com/DaehwanKimLab/hisat2, accessed on 17 October 2025), followed by bowtie2 [14] (V2.3.5, https://github.com/BenLangmead/bowtie2, accessed on 17 October 2025), which aligned the post-quality-control sequence to the reference transcript sequence. RSEM [15] (V1.3.1, https://github.com/deweylab/RSEM, accessed on 17 October 2025) was utilized to quantify raw read counts for each gene. FPKM values (fragments per kilobase per million bases) were subsequently calculated from these counts for visualization purposes only. To detect differentially expressed genes (DEGs) between groups, differential expression analysis was performed using DESeq2 [16] (V1.22.2, https://github.com/thelovelab/DESeq2, accessed on 17 October 2025) on the raw count data. Size factors for normalization were estimated using the median-of-ratios method implemented in DESeq2. Genes that satisfied the following criteria were designated as differentially expressed: a screening threshold of |log_2_FC(fold change)| ≥ 1 and a *p*-adjust ≤ 0.05. The reported *p*-adjust were already false-discovery-rate adjusted by the Benjamini–Hochberg procedure implemented in DESeq2, and genes with *p*-adjust ≤ 0.05 were considered statistically differentially expressed.

### 2.4. Bioinformatics Analysis

Gene Ontology (GO) is categorized into three ontologies: molecular function, biological process, and cellular component. GO enrichment analysis was performed using Goseq [17] (V1.22, https://github.com/lmika/goseq, accessed on 20 October 2025) and GO terms with *p* ≤ 0.05 were selected as significantly enriched GO entries. The Kyoto Encyclopedia of Genes and Genomes (KEGG), the primary public database on pathways, was annotated using KOBAS [18] (V3.0, https://github.com/xmao/kobas, accessed on 20 October 2025). The pathways that exhibited a *p*-value less than 0.05 were designated as pathways that were significantly enriched in differentially expressed genes. Protein–Protein Interaction (PPI) Network Analysis was executed via the STRING database [19] (v2.2.1, https://cn.string-db.org/, accessed on 20 October 2025) and utilizing Cytoscape software (v3.8.1, https://cytoscape.org/, accessed on 20 October 2025) for visualization. The cytoHubba plugin was employed to calculate degree centrality for each node, and proteins with degree ≥ 5 were selected for network visualization. We constructed protein–protein interaction (PPI) networks of differentially expressed genes.

### 2.5. Reverse Transcription-Quantitative Real-Time PCR (RT-qPCR)

To verify the accuracy of the sequencing results, six differentially expressed genes (DEGs) in the PPI network were randomly selected for validation using quantitative real-time polymerase chain reaction (RT-qPCR). Total RNA was reverse transcribed into cDNA using the ToloScript RT EasyMix for qPCR (with 2-Step gDNA Erase-Out) kit.

RT-qPCR was performed using the 2 × Q3 SYBR qPCR Master Mix (Universal) kit on the Bio-Rad CFX96 real-time detection system (BioRad, Hercules, CA, USA). The 20 μL quantitative PCR reaction mixture consisted of 10 μL of SYBR Green master mix, 1 μL of cDNA template, 0.8 μL each of forward and reverse primers, and 8.4 μL of nuclease-free ddH_2_O.

The RT-qPCR amplification protocol consisted of predenaturation at 95 °C for 30 s, followed by 40 cycles, hot start at 95 °C for 10 s, hot start at 56 °C for 30 s, and hot start at 72 °C for 30 s. The relative expression levels of the genes were calculated using the 2^−ΔΔCt^ method, using β-actin as the internal control. All primer sequences are listed in Table 1.

### 2.6. Statistical Analysis

Statistical analysis was performed using GraphPad Prism (v5.0, La Jolla, CA, USA). Data are presented as mean ± standard error of the mean (SEM). Between-group differences were evaluated by Student’s *t*-test; significance was defined as *p* < 0.05.

## 3. Results

### 3.1. RNA Sequencing Data Summary

After completion of RNA-seq, the original sequencing data were filtered to the linker sequence, etc., the high parity group and the low parity group obtained an average of 55,980,603 clean reads, Q20 and Q30 of each sample were greater than 97% and 92%, GC content greater than 47%, the above results are shown in Table 2, the sequencing results have high reliability, can be used for subsequent analysis.

### 3.2. Identification and Analysis of DEGs

**Figure 1:**
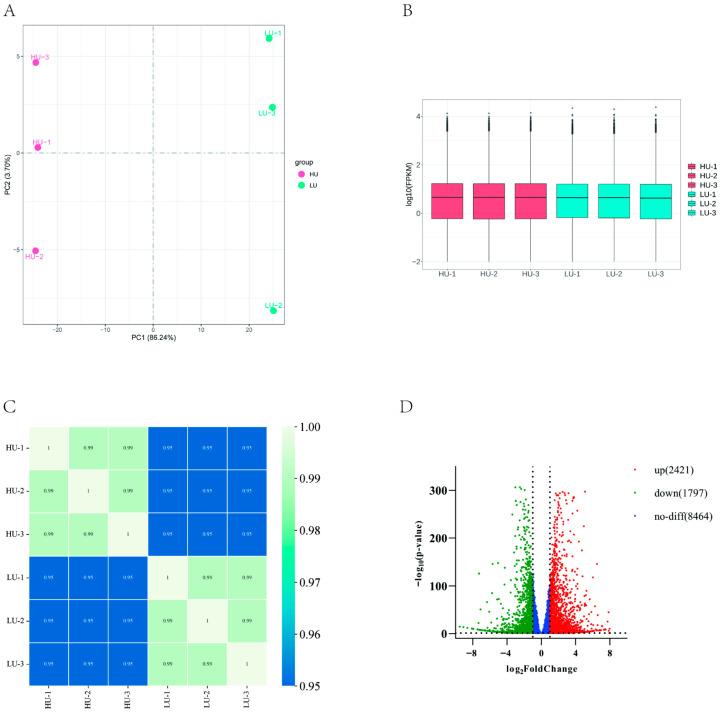
Screening of DEGs in the fallopian tubes of high and low parity sows. (**A**) PCA diagrams of all gene expression in the two groups of samples. (**B**) Box line diagrams showing mRNA expression characteristics. (**C**) Heat diagrams of mRNA. (**D**) Volcanic diagrams showing mRNA up-regulation and down-regulation.

Principal component analysis (PCA) of oviduct transcriptome data from Wanyue black pigs revealed clear separation between high- and low-parity groups along PC1, which accounted for 86.24% of variance (Figure 1A), indicating distinct global expression profiles. Following FPKM normalization, expression distribution analysis showed consistent intra-group levels with inter-group variation (Figure 1B), while correlation heatmaps confirmed strong intra-group but weaker inter-group correlations (Figure 1C). Differential expression analysis using DESeq2 identified 4218 DEGs (2421 up- and 1797 down-regulated) under thresholds of |log_2_FC| ≥ 1 and adjusted *p* < 0.05 (Figure 1D).

### 3.3. GO Enrichment Analysis of Differential Genes

**Figure 2:**
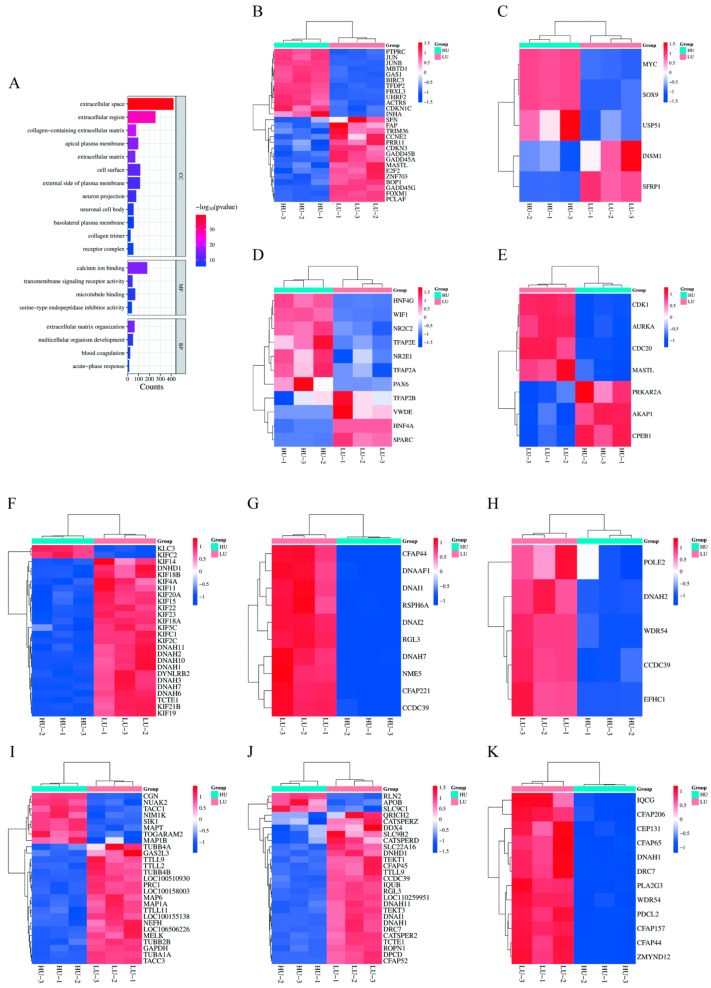
GO analysis of DEGs in the fallopian tubes of high and low parity sows. (**A**) The first 20 enriched GO terms of DEGs. The abscissa represents the number of genes enriched, and the ordinate represents the GO name. (**B**) Gene expression pattern diagram of cell cycle regulation. (**C**) Gene expression pattern diagram of cell cycle process regulation. (**D**) Gene expression pattern diagram of anatomical structure development. (**E**) Gene expression pattern diagram of positive regulation of the meiotic cell cycle process during oocyte maturation. (**F**) Gene expression pattern diagram of microtubule-driven movement. (**G**) Gene expression pattern diagram of cilia movement. (**H**) Gene expression pattern diagram of cilia-dependent cell movement. (**I**) Gene expression pattern diagram of microtubule cytoskeleton. (**J**) Gene expression pattern diagram of sperm flagella movement. (**K**) Gene expression pattern diagram of sperm axis filament assembly.

Gene ontology enrichment analysis of 4218 DEGs identified 276 significantly enriched terms in biological processes, 78 in cellular components, and 103 in molecular functions (*p* < 0.05). Key enriched terms related to reproduction included “sperm flagellum”, “flagellated sperm motility”, and “positive regulation of meiotic cell cycle” (Table 3, Figure 2). Expression heatmaps revealed that genes such as *JUNB*, *MYC*, and *CDK1* were upregulated in the high-parity group within processes including cell cycle regulation. Conversely, genes including *KIF14*, *DNAI1*, and *DNAH2* were upregulated in the low-parity group in functions associated with microtubule and cilia motility (Figure 2B–K).

### 3.4. KEGG Enrichment Analysis of Differential Genes

**Figure 3:**
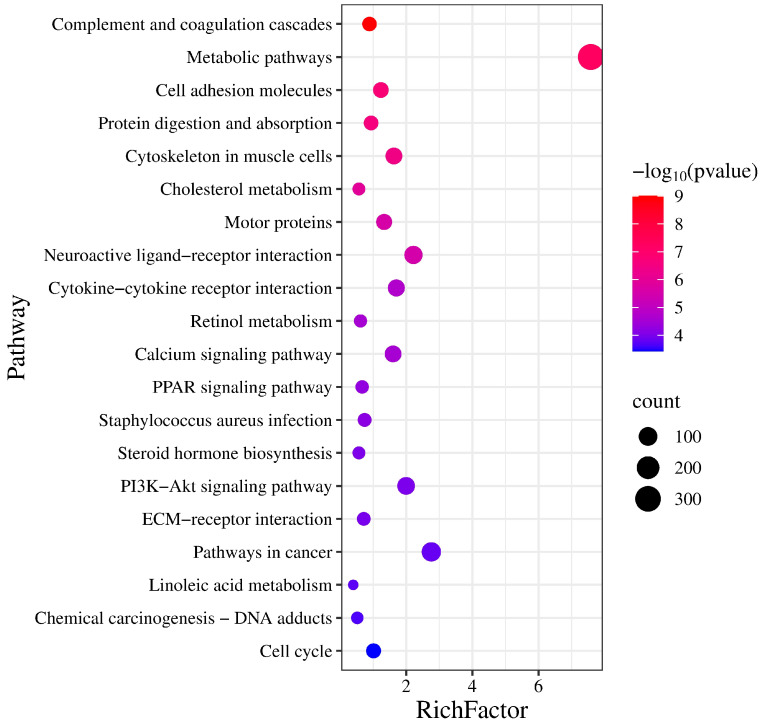
KEGG pathway enrichment analysis of differentially expressed genes. The abscissa indicates the enrichment factor, and the ordinate lists the pathway names.

**Figure 4:**
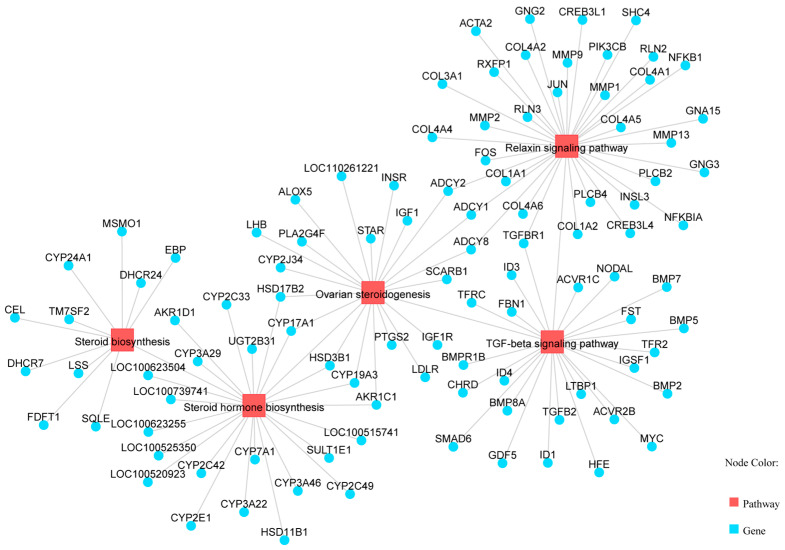
Pathways related to estrogen synthesis, including steroid hormone biosynthesis pathway, ovarian steroid generation pathway, steroid biosynthesis pathway, relaxin signaling pathway, and TGF-β signaling pathway.

**Figure 5:**
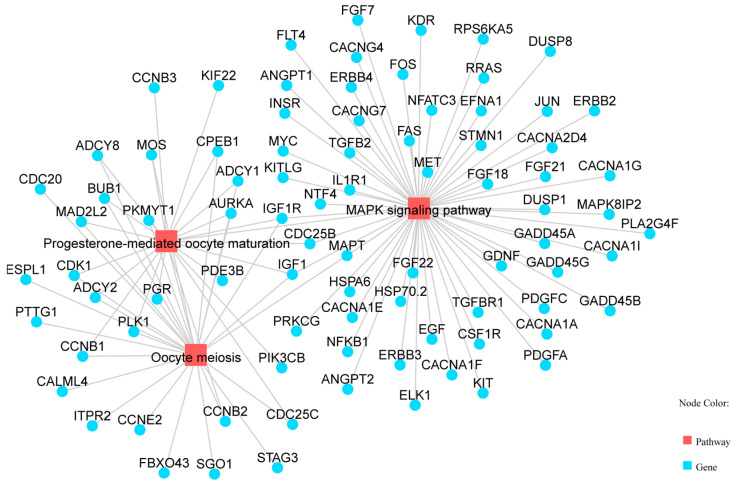
Pathways related to oocyte meiosis, including oocyte meiosis, progesterone-mediated oocyte maturation, and MAPK signaling pathway.

KEGG pathway enrichment analysis identified 89 significantly enriched pathways (*p* < 0.05). Several pathways were related to reproduction, including “Steroid hormone biosynthesis”, “Ovarian steroidogenesis”, “Steroid biosynthesis”, and the “Relaxin signaling pathway” (Table 4, Figure 3). Five genes (*AKR1C1*, *HSD3B1*, *CYP19A3*, *CYP17A1*, and *HSD17B2*) were enriched in two steroidogenesis-related pathways. In the network of these pathways, genes such as HSD3B1, *CYP17A1*, *STAR*, *RXFP1*, and *TGFBR1* interact to regulate hormone synthesis (Figure 4). In pathways of oocyte meiosis and maturation, genes including *AURKA*, *CDK1*, *CDC20*, *CCNB1*, and *PGR* were centrally regulated (Figure 5).

### 3.5. PPI Network Analysis

**Figure 6:**
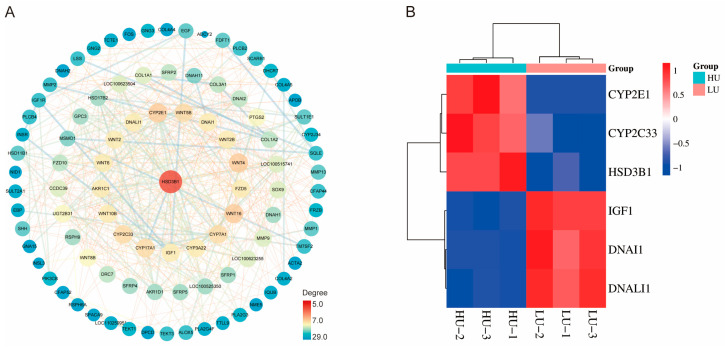
A protein–protein interaction (PPI) network was constructed from the differentially expressed genes (DEGs), followed by screening to identify key genes. (**A**) The resultant PPI network. (**B**) Heatmap depicting the expression profiles of the key genes.

To identify key genes regulating oviduct function in high- and low-parity Wanyue black pigs, a protein–protein interaction (PPI) network was constructed based on differentially expressed genes (DEGs). The network (Figure 6A) displays protein nodes with an interaction degree ≥ 5, where node size and color (blue to red) reflect connectivity and interaction strength, respectively. *HSD3B1* occupied a central position due to its high connectivity, suggesting its importance in oviduct physiology across parities. Other key genes, including *DNAI1* and *DNALI1*, also showed notable functional associations. Furthermore, a heatmap (Figure 6B) illustrates differential expression of these genes between high- and low-parity sows. These analyses highlight pivotal genes and their interactions potentially involved in reproductive regulation.

### 3.6. RNA-Seq Data Validation by RT-qPCR

**Figure 7:**
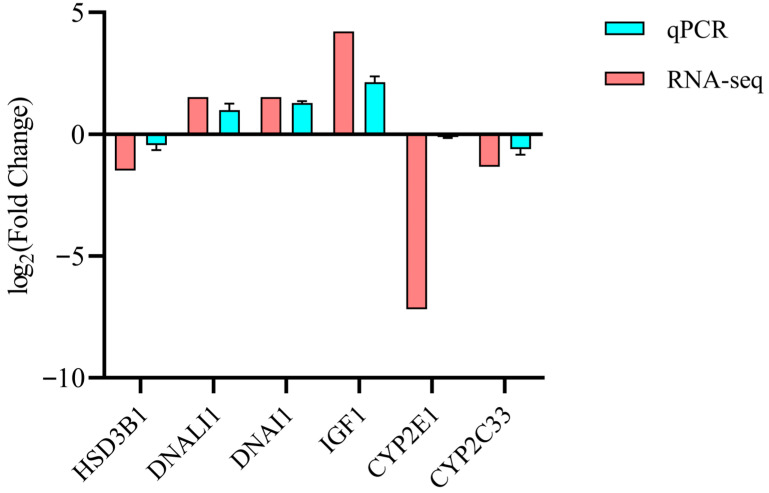
The expression of differentially expressed genes (DEGs) was validated using RT-qPCR (*n* = 3). The HU group served as the reference, with gene expression in the LU group presented as log_2_ (Fold Change).

To validate RNA-seq data, six differentially expressed genes (DEGs) were randomly selected for confirmatory RT-qPCR analysis in oviducts from high (HU) and low (LU) fertility sows (*n* = 6 per group). Expression patterns of all six DEGs were concordant with RNA-seq findings (Figure 7), confirming the reliability of the transcriptomic results.

## 4. Discussion

In this study, we systematically compared the transcriptome differences between the oviducts of sows from high- and low-parity Wanyue black pigs. We employed RNA-seq technology to achieve this objective, and our analysis identified 4218 differentially expressed genes (DEGs), of which 2421 were up-regulated genes and 1797 were down-regulated genes. The genes in question were predominantly enriched in pathways associated with reproductive functions, including oocyte maturation, steroid hormone synthesis, and cilia movement. This finding suggests that sows with different parities may influence their reproductive ability by regulating the expression of relevant genes in the oviduct. To verify the reliability of the RNA-seq data, six candidate genes were selected for qPCR validation, and the expression trends were consistent with the transcriptome data. This confirmed that the results of this study had a high degree of confidence. These results demonstrate that parity induces transcriptional reprogramming in the oviduct, shifting its functional emphasis from gamete transport toward microenvironmental support for maturation. This provides a molecular basis for optimizing parity-specific reproductive management.

Gene Ontology (GO) enrichment analysis revealed the upregulated expression of cell cycle regulatory and oocyte maturation-associated genes, including *JUNB*, *MYC*, *SOX9*, and *CPEB1*, in the oviductal tissues of the high-parity cohort. This transcriptional activation facilitates cellular proliferation [20] and promotes oocyte meiotic progression [21], thereby establishing a molecular milieu conducive to oocyte development and ovulation. Furthermore, elevated expression of *PAX6* and *TFAP2A* in high-parity sows may contribute to oviductal structural integrity and optimization of the local reproductive tract microenvironment [22,23].

Comprehensive pathway enrichment analysis revealed that differentially expressed genes (DEGs) function coordinately within specific signaling cascades, establishing a synergistic regulatory network that modulates oviductal reproductive support functions across multiple molecular levels. Within the “steroid hormone biosynthesis” and “ovarian steroidogenesis” pathways, pivotal genes including *HSD3B1*, *CYP17A1*, and *STAR* exhibited significant upregulation in the high-parity cohort. The encoded enzymes represent key rate-limiting factors in progesterone and estrogen synthesis [24,25]. This transcriptional activation implies enhanced local steroidogenic capacity within the oviductal microenvironment of high-parity sows, which may directly modulate epithelial secretory activity, ciliary beat frequency, and smooth muscle contractility. Consequently, this hormonal microenvironment adaptation generates optimal conditions for early embryonic survival and development, potentially representing an adaptive molecular mechanism for embryo viability maintenance in high-parity sows [26]. Within the “oocyte meiosis” and “progesterone-mediated oocyte maturation” pathways, differential expression of *AURKA*, *CDK1*, *CDC20*, and *PGR* constitutes a refined regulatory module. *AURKA* and *CDK1* function as core kinases governing meiotic spindle assembly and chromosome segregation [27,28], whereas *PGR*, as the progesterone receptor, transduces hormonal signals to orchestrate meiotic resumption [29]. The coordinated upregulation of these genes in high-parity sows suggests that the oviduct may provide enhanced paracrine molecular support for final oocyte maturation and chromosomal stability, thereby safeguarding oocyte quality and fertilization competence. Regarding ciliary motility and sperm flagellar assembly, genes encoding core axonemal dynein complex components—including *DNAI1*, *CFAP44*, and *IQCG*—were significantly enriched and upregulated in the low-parity group [30,31,32]. Their elevated expression directly correlates with ciliary beat efficiency, implying that low-parity sows possess enhanced oviductal ciliary motility dynamics. This molecular advantage facilitates more efficient gamete transport, sperm–egg fusion, and early embryonic transit to the uterus, thereby providing a mechanistic explanation for the typically higher conception rates observed in low-parity sows.

These pathways exhibit intricate crosstalk. For instance, steroidogenic pathway products such as progesterone serve as signaling molecules that activate *PGR*, thereby modulating downstream MAPK signaling and regulating oocyte maturation [33]. Concurrently, the hormonal milieu governs the expression of genes associated with ciliary motility. Hub genes identified through PPI network analysis in the current study (e.g., *HSD3B1* and *DNAI1*) occupy critical nodes at these pathway intersections, suggesting that parity-associated effects on reproductive function may be mediated through coordinated regulation of a core hub gene set, thereby systematically modifying oviductal functional states spanning steroidogenesis, oocyte support, and transport. Furthermore, consistent with existing literature, the decline in reproductive performance observed in high-parity sows may also be associated with accumulated oxidative stress, dysregulated apoptosis, and altered autophagy. The differentially expressed genes and pathways identified herein thus provide novel molecular targets and insights for elucidating these complex mechanisms.

Piñán J et al. [4] documented that Iberian sows achieve maximal reproductive performance at parities 2–4, with subsequent decline beyond parity 5, a pattern congruent with the functional divergence observed at extreme parity levels in the present study. Thomas et al. [5] similarly demonstrated that multiparous sows exhibit superior total parity sizes compared to their primiparous counterparts, reinforcing the profound impact of parity on reproductive efficiency. Notably, these investigations focused exclusively on phenotypic manifestations, whereas transcriptomic analysis reveals the underlying tissue-specific mechanisms. Specifically, the elevated expression of the ciliary motility-associated gene DNAI1 in low-parity sows aligns with the postulation by Teijeiro et al. [9] regarding optimized oviductal transport function during early reproductive cycles. Moreover, this investigation establishes a novel paradigm that the oviduct functions not as a static conduit but as a parity-sensitive regulatory organ. Complementing this, Nuntapaitoon et al. [7] demonstrated that parity modulates immunoglobulin G (IgG) concentrations in sow colostrum, reflecting systemic immune adaptation to reproductive history, whereas these findings elucidate parity-induced localized oviductal modifications occurring independently of systemic immune function.

This transcriptomic analysis of Wanyue Black pig oviducts reveals parity-related differences in gene expression; however, two primary limitations warrant consideration. First, the sample size was modest (*n* = 3 per group), constraining statistical power and generalizability. Second, the absence of concurrent phenotypic data—such as fertilization rates, embryo quality, or tubal motility measurements—precludes direct validation of how the observed transcriptomic shifts translate to reproductive performance. It should be emphasized that these transcriptomic profiles reflect functional specialization rather than superiority: low-parity oviducts prioritize efficient sperm transport through enhanced ciliary motility, whereas high-parity oviducts allocate resources toward steroid synthesis and oocyte maturation support, representing parity-appropriate physiological adaptations. Future studies should expand cohort sizes and integrate functional phenotyping to confirm these bioinformatics-derived mechanisms.

## 5. Conclusions

This study employed RNA-seq to compare oviductal transcriptomes between high-parity (9 parities) and low-parity (1 parity) Wanyue Black pigs, revealing significant parity-driven reprogramming of reproductive function. High parity upregulated genes governing oocyte maturation and steroidogenesis—including JUNB, MYC, and HSD3B1—whereas low parity enhanced expression of sperm motility genes such as DNAI1, CFAP44, and IQCG, indicating a functional shift from gamete transport to oocyte microenvironment support. PPI network analysis positioned HSD3B1 and DNAI1 as central regulators, and qRT-PCR validation confirmed transcriptomic reliability. These findings establish a molecular framework for parity-specific reproductive management in indigenous pig breeds and underscore the need for breed-tailored strategies. Future research should expand sample sizes, integrate functional phenotyping, and employ single-cell profiling to translate these mechanisms into precision breeding applications.

## Figures and Tables

**Table 1:** Primer sequences used for RT-qPCR.

Gene Name	Primer (5′—3′)	Product Size (bp)
*β-actin*	F: GGACTTCGAGCAGGAGATGG	138
	R: AGGAAGGAGGGCTGGAAGAG	
*HSD3B1*	F: TGAAAGGTACCCAGCTCCTG	112
	R: TGGATGACCTCCCTGTAGGA	
*DNALI1*	F: ACAGAGAAGCGGGAAAGTGA	88
	R: GCTGCTGATTGGTCCTCTTG	
*DNAI1*	F: AGTCCGTCAAGGTGGTGATT	120
	R: CAGCCACTTTCTCAGATGCC	
*IGF1*	F: TTCAACAAGCCCACAGGGTA	102
	R: CTCCAGCCTCCTCAGATCAC	
*CYP2E1*	F: AATCCCTGCCATCAAGGACA	94
	R: CAGGTTGGAGGGAATGAGGT	
*CYP2C33*	F: AGCCCTTTGACCCTACCTTC	87
	R: TGTCGTAGTGGAAACGGTCA	

**Table 2:** Sample RNA-seq data statistics.

Sample	Clean Reads	Q20 (%)	Q30 (%)	GC (%)
HU1	53,822,610	98.52	94.71	48.08
HU2	56,101,360	98.47	94.48	47.89
HU3	57,056,748	97.91	93.08	47.92
LU1	53,460,190	98.46	94.51	48.50
LU2	53,248,756	98.53	94.78	48.61
LU3	62,193,952	98.53	94.77	48.60

**Table 3:** GO terms related to reproduction.

GO Terms	*p*-Value	Gene Count	Gene
flagellated sperm motility	0.0000016	27	*DNAH1*, *DRC7*, *CATSPER2*, *DDX4*, *DNHD1*, *SLC9C1*, *DPCD*, *LOC110259951*, *SLC22A16*, *IQUB*, *TEKT1*, *TEKT3*, *APOB*, *DNAI1*, *CFAP45*, *CCDC39*, *DNAH11*, *CFAP52*, *CATSPERZ*, *TCTE1*, *SLC9B2*, *CATSPERD*, *RLN2*, *RGL3*, *QRICH2*, *ROPN1*, *TTLL9*
sperm axoneme assembly	0.0074467	12	*PDCL2*, *DNAH1*, *DRC7*, *CEP131*, *PLA2G3*, *CFAP206*, *CFAP65*, *CFAP157*, *ZMYND12*, *IQCG*, *WDR54*, *CFAP44*
positive regulation of meiotic cell cycle process involved in oocyte maturation	0.0012398	7	*CDC20*, *CPEB1*, *PRKAR2A*, *CDK1*, *MASTL*, *AKAP1*, *AURKA*

**Table 4:** KEGG pathway related to reproduction.

KEGG Pathway	*p*-Value	Gene Count	Gene
Steroid hormone biosynthesis	0.00009	23	*UGT2B31*, *LOC100739741*, *AKR1C1*, *HSD3B1*, *AKR1D1*, *CYP3A22*, *LOC100525350*, *CYP3A46*, *CYP19A3*, *LOC100515741*, *CYP7A1*, *CYP17A1*, *LOC100623255*, *CYP2C49*, *CYP3A29*, *HSD11B1*, *LOC100623504*, *CYP2C33*, *SULT1E1*, *LOC100520923*, *CYP2C42*, *HSD17B2*, *CYP2E1*
Ovarian steroidogenesis	0.00041	20	*SCARB1*, *PLA2G4F*, *AKR1C1*, *INSR*, *HSD3B1*, *LHB*, *ADCY2*, *CYP19A3*, *IGF1*, *ADCY1*, *ADCY8*, *PTGS2*, *IGF1R*, *CYP17A1*, *CYP2J34*, *STAR*, *ALOX5*, *HSD17B2*, *LDLR*, *LOC110261221*
Steroid biosynthesis	0.00151	10	*SQLE*, *EBP*, *CYP24A1*, *MSMO1*, *DHCR24*, *DHCR7*, *CEL*, *LSS*, *FDFT1*, *TM7SF2*
Relaxin signaling pathway	0.00155	34	*SHC4*, *ADCY2*, *ADCY1*, *PIK3CB*, *ADCY8*, *RXFP1*, *GNG3*, *GNA15*, *GNG2*, *CREB3L4*, *CREB3L1*, *INSL3*, *JUN*, *MMP1*, *MMP2*, *FOS*, *MMP9*, *NFKB1*, *TGFBR1*, *COL1A1*, *ACTA2*, *NFKBIA*, *COL3A1*, *PLCB4*, *COL1A2*, *MMP13*, *COL4A2*, *COL4A1*, *COL4A4*, *COL4A6*, *RLN2*, *COL4A5*, *RLN3*, *PLCB2*

## Data Availability

The original data presented in the study are openly available online repositories. The names of the repository/repositories and accession number(s) can found in NCBI BioProject PRJNA1299546.
